# Cardiac Lymphatic Dysfunction in Heart Failure: A New Paradigm for Congestion, Inflammation, and Therapy

**DOI:** 10.3390/medsci14020266

**Published:** 2026-05-20

**Authors:** Francisco Epelde

**Affiliations:** 1 Internal Medicine Department, Parc Taulí Hospital Universitari, Institut d’Investigació i Innovació Parc Taulí (I3PT-CERCA), Universitat Autònoma de Barcelona, 08208 Sabadell, Spain; fepelde@tauli.cat; 2 APTIMA Centre Clinic, Mutua de Terrassa, 08221 Terrassa, Spain

**Keywords:** heart failure, cardiac lymphatics, myocardial edema, inflammation, fibrosis

## Abstract

**Background:** Heart failure (HF) has traditionally been interpreted through hemodynamic, neurohormonal, and cardiorenal frameworks. Although these models explain many aspects of clinical decompensation, they do not fully account for persistent tissue congestion, unresolved myocardial edema, chronic sterile inflammation, and progressive fibrosis despite optimized therapy. **Objectives:** To review the anatomy, physiology, and pathobiological relevance of the cardiac lymphatic system in HF and to evaluate whether cardiac lymphatic dysfunction constitutes a mechanistic bridge linking congestion, inflammation, and adverse remodeling. Methods: This narrative review was based on a structured literature search of PubMed/MEDLINE, supplemented by manual backward reference screening and bibliographic verification through journal webpages. The search covered January 2000 to 15 April 2026, with emphasis on 2018 onward and on seminal mechanistic studies. Search domains included cardiac lymphatics, heart failure, lymphangiogenesis, myocardial edema, congestion, inflammation, myocardial infarction, pressure overload, and HFpEF. **Results:** Cardiac lymphatics regulate myocardial clearance of interstitial fluid, proteins, cytokines, lipids, and immune cells. Preclinical experimental evidence, mainly derived from myocardial infarction, pressure-overload, and lymphatic-insufficiency models, indicates that impaired lymphatic transport or insufficient lymphangiogenic adaptation promotes myocardial edema, inflammatory persistence, fibroblast activation, collagen deposition, and ventricular dysfunction. Human observational and early translational studies suggest that lymphatic dysregulation may also be relevant in selected HF phenotypes, although direct clinical evidence remains limited. Conversely, lymphangiogenic and lymphatic-restorative strategies, especially through the VEGF-C/VEGFR-3 axis, reduce edema, enhance inflammatory resolution, attenuate fibrosis, and improve ventricular performance in preclinical models. **Conclusions:** Cardiac lymphatic dysfunction provides a compelling conceptual framework that links congestion and inflammation in HF. Rather than acting as a passive bystander, the cardiac lymphatic circulation appears to be an active determinant of myocardial homeostasis and disease progression. Recognition of lymphatic insufficiency as a pathogenic component of HF may open new diagnostic and therapeutic avenues, including tissue-focused decongestion, lymphatic phenotyping, and targeted lymphatic repair.

## 1. Introduction

Heart failure remains one of the leading causes of hospitalization, disability, and cardiovascular mortality worldwide despite major advances in pharmacological and device-based therapy [1,2,3]. Contemporary HF biology integrates abnormalities in ventricular structure and function with neurohormonal activation, renal sodium retention, endothelial dysfunction, and systemic hemodynamic stress [4,5,6]. Even so, several clinically relevant features of HF remain incompletely explained by conventional models. Among these are persistent congestion despite apparently adequate intravascular decongestion, chronic low-grade inflammation that persists well beyond the initiating insult, and progressive fibrosis despite guideline-directed treatment [7,8,9,10,11,12].

These unresolved features are central to clinical outcome. Congestion remains the dominant cause of HF hospitalization and a strong predictor of recurrent decompensation and death [4,5,6,7,8,9]. Chronic inflammation contributes to cardiomyocyte dysfunction, endothelial activation, fibroblast stimulation, and extracellular matrix turnover [10,11,12]. Together, these observations suggest that the failing heart cannot be understood solely as a pressure-overloaded or neurohormonally activated pump. It is also an organ with disturbed tissue homeostasis.

The lymphatic circulation offers a biologically coherent framework to address this gap. The lymphatic system is essential for interstitial fluid balance, removal of proteins and macromolecules, immune-cell trafficking, and inflammatory resolution [13,14,15,16,17,18]. In the heart, lymphatic vessels drain excess fluid, cytokines, lipids, and immune cells from the myocardium. Failure of this system may therefore contribute directly to myocardial edema, inflammatory persistence, extracellular matrix expansion, and chamber dysfunction [19,20,21].

This concept is particularly relevant because the myocardium is highly sensitive to abnormalities in the interstitial compartment. Even modest increases in myocardial water content can alter compliance, oxygen diffusion, cell–matrix interactions, and capillary performance [22,23]. Likewise, inadequate clearance of leukocytes, cytokines, and matrix fragments can transform reparative inflammation into chronic fibro-inflammatory remodeling [24]. Cardiac lymphatic dysfunction may therefore represent a missing mechanistic link between congestion and inflammation.

Recent work has substantially changed the field. Preclinical experimental studies, including myocardial infarction, pressure-overload, and direct lymphatic-insufficiency models, have shown that cardiac lymphangiogenesis is activated after injury but often remains insufficient or dysfunctional, and that impaired lymphatic drainage worsens edema, fibrosis, and ventricular dysfunction [25,26,27,28,29,30,31]. Human observational, translational, and early feasibility studies further suggest that lymphatic dysregulation may be relevant in symptomatic HF, although the clinical evidence remains limited and hypothesis-generating [32,33,34,35]. This review examines cardiac lymphatic dysfunction as a new paradigm in HF and discusses its implications for pathophysiology, phenotyping, and therapy.

## 2. Methods of Literature Search and Bibliographic Extraction

This narrative review was prepared using a structured literature search designed to maximize bibliographic reliability and mechanistic relevance. PubMed/MEDLINE served as the primary database. The search was supplemented by backward screening of references from key reviews and landmark studies. Bibliographic details were checked against journal webpages when needed to confirm journal title, year, volume, pagination, and DOI.

The search covered 1 January 2000 to 15 April 2026. Particular emphasis was placed on literature published from 2018 onward and on seminal earlier articles required to contextualize myocardial edema, lymphatic biology, inflammatory remodeling, and HF pathophysiology [36,37,38,39,40]. Search terms included “cardiac lymphatics,” “heart failure,” “cardiac lymphatic dysfunction,” “lymphangiogenesis,” “myocardial edema,” “interstitial congestion,” “VEGF-C,” “VEGFR-3,” “myocardial infarction,” “pressure overload,” “fibrosis,” “inflammation,” “HFpEF,” and “lymphatic drainage.”

Eligible publications included original experimental studies, translational studies, pathology studies, human observational reports, feasibility studies, and high-impact reviews directly relevant to one or more of the following domains: cardiac lymphatic anatomy and development; interstitial fluid regulation and myocardial edema; immune-lymphatic interactions; fibrosis and remodeling; HF phenotypes; and lymphatic-targeted diagnostics or therapies [19,20,21,24,36,37,38,39,40]. References were selected based on thematic fit, methodological importance, translational value, and bibliographic certainty.

In the narrative synthesis, the available evidence was interpreted according to its source and translational level, distinguishing preclinical experimental studies, human observational or translational studies, and early clinical feasibility data.

## 3. Cardiac Lymphatic Anatomy, Development, and Physiological Function

The lymphatic vasculature is a specialized low-pressure network that maintains interstitial fluid balance and returns proteins, lipids, immune cells, and macromolecules to the venous circulation [13,14,15,16,17,18]. Initial lymphatic capillaries are characterized by permeable endothelial architecture, discontinuous basement membranes, and overlapping junctions that facilitate entry of fluid and cells when interstitial pressure rises. These capillaries merge into pre-collectors and collecting vessels containing valves and contractile elements that support directional flow [14,17].

**Figure 1:**
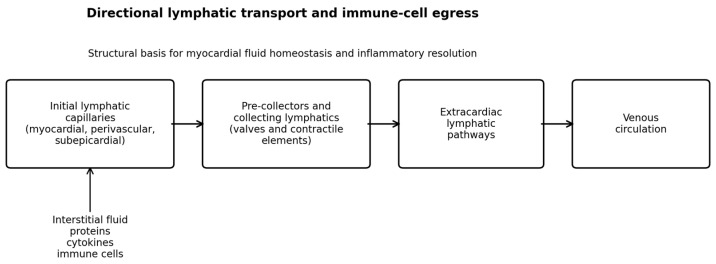
Schematic representation of cardiac lymphatic anatomy. Initial lymphatic capillaries located in the myocardial, perivascular, and subepicardial compartments collect interstitial fluid, proteins, immune cells, and inflammatory mediators. These vessels converge into larger collecting lymphatics with valves and contractile elements, supporting directional flow toward extracardiac lymphatic pathways and the venous circulation. This anatomical organization provides the structural basis for myocardial fluid homeostasis, immune-cell trafficking, and inflammatory resolution.

In the heart, lymphatic vessels form a structured network within epicardial, peri-coronary, and myocardial compartments [36,37,38,39,40]. Classical morphologic work documented substantial remodeling of myocardial lymphatics in terminal HF [37]. More recent developmental studies demonstrated that cardiac lymphatics are heterogeneous in origin and dynamically respond to injury [38,39]. This heterogeneity may have pathophysiological consequences because different cardiac territories may differ in lymphatic reserve and reparative plasticity. Schematic representation of cardiac lymphatic anatomy is presented in Figure 1.

Cardiac lymphatics perform several interrelated functions. First, they regulate myocardial interstitial fluid balance by removing ultrafiltrate generated by coronary microvascular exchange [19,36,40,41]. Second, they clear plasma proteins and macromolecules that cannot efficiently return through venous capillary pathways [13,17,41]. Third, they mediate immune-cell trafficking and contribute to the clearance of cytokines, cellular debris, and inflammatory mediators [15,16,24,42]. Fourth, by shaping the biochemical composition of the interstitium, they indirectly influence fibroblast activation, matrix turnover, and tissue repair [19,24,40].

These functions are particularly important because the myocardium is an organ in which small perturbations of the extracellular compartment can have disproportionate mechanical consequences. Unlike more compliant tissues, the heart depends on tightly controlled interstitial conditions to preserve ventricular compliance, capillary exchange, and diffusion efficiency. The cardiac lymphatic circulation should therefore be regarded as a central component of myocardial homeostasis rather than a minor accessory system.

## 4. Myocardial Edema as a Pathogenic Process

Myocardial edema is increasingly recognized as a biologically active component of cardiac disease rather than a passive marker of injury [22,23,43]. Edema can increase tissue pressure, compress microvessels, lengthen diffusion distance for oxygen, distort matrix geometry, and impair both diastolic and systolic performance [22,23]. In addition, an edematous interstitium retains proteins, cytokines, alarmins, and leukocytes, making edema both a biomechanical and an inflammatory process.

The lymphatic circulation is central to edema resolution. Under physiological conditions, lymphatic transport increases when interstitial load rises. In disease, however, increased microvascular filtration may overwhelm lymphatic reserve, especially when elevated venous pressure simultaneously impairs lymph return [17,19,33,41]. This creates a setting in which tissue congestion can persist despite reductions in intravascular volume. Classic preclinical experimental work supports the pathogenic relevance of this mechanism. Chronic interruption of cardiac lymph flow increased collagen synthesis and interstitial fibrosis in rabbits, directly linking lymphatic dysfunction to structural remodeling [44]. More broadly, monocyte- and macrophage-driven inflammatory repair programs after myocardial injury provide a biologically plausible context in which defective lymphatic clearance may become maladaptive rather than restorative [45].

## 5. Cardiac Lymphatics in Myocardial Infarction and Post-Infarction Remodeling

Myocardial infarction is the best-characterized setting in which cardiac lymphatics have emerged as relevant regulators of healing. MI causes abrupt cardiomyocyte death, microvascular injury, interstitial edema, and a robust inflammatory response [11,45]. Effective repair requires both sufficient immune activation and timely inflammatory resolution. Cardiac lymphatics appear to contribute to both processes.

In experimental MI models, endogenous lymphangiogenesis is activated after injury but is often insufficient relative to tissue demand [25,26,38,46,47,48]. Henri et al. demonstrated that selective stimulation of cardiac lymphangiogenesis reduced myocardial edema and fibrosis and improved ventricular function after MI [25]. Vieira et al. subsequently showed that the cardiac lymphatic system stimulates resolution of inflammation following MI and that enhanced lymphangiogenesis improves immune-cell clearance and healing [26]. Houssari et al. further showed that lymphatic and immune-cell cross-talk regulates cardiac recovery after experimental MI [27].

Subsequent preclinical work extended these findings mechanistically. Trincot et al. identified adrenomedullin as an inducer of cardiac lymphangiogenesis after MI and a regulator of edema through connexin 43-associated signaling [28]. Monaghan et al. and Berkeley et al. placed VEGFR3 signaling and lymphatic regeneration within a broader framework of myocardial repair [47,48]. Glinton et al. showed that macrophage-derived VEGF-C induced by efferocytosis ameliorates cardiac injury and inflammation, thereby linking phagocytic resolution to reparative lymphatic signaling [49]. Not all preclinical studies point in exactly the same direction, however. Keller et al. reported that genetic blockade of lymphangiogenesis does not necessarily impair cardiac function after MI, emphasizing that the functional impact of lymphatic remodeling may depend on model, injury size, and timing [50].

Taken together, the evidence indicates that cardiac lymphatics are meaningful modifiers of infarct healing. They influence edema resolution, leukocyte clearance, fibrotic remodeling, and eventually ventricular performance.

## 6. Pressure Overload and Chronic Hemodynamic Stress

Pressure-overload HF provides a complementary model of lymphatic maladaptation. Chronic pressure stress induces hypertrophy, microvascular strain, inflammation, and extracellular matrix expansion [12]. In response, the lymphatic system undergoes remodeling that appears adaptive but may remain functionally incomplete.

In preclinical pressure-overload models, Heron et al. demonstrated that endogenous cardiac lymphangiogenesis limits inflammation and perivascular fibrosis and delays HF development in pressure-overload remodeling [29]. Lin et al. showed that the VEGF-C/VEGFR-3 axis protects against pressure-overload-induced cardiac dysfunction through regulation of lymphangiogenesis [30]. These findings suggest that lymphatic expansion is part of the heart’s intrinsic attempt to adapt to chronic stress.

However, increased lymphatic density is not synonymous with restored drainage. Bizou et al. found that cardiac macrophage subsets differentially regulate lymphatic network remodeling during pressure overload [51]. This implies that inflammatory context shapes not only the quantity but also the quality of lymphatic adaptation. A remodeled lymphatic network may therefore be anatomically expanded but functionally inadequate.

## 7. Cardiac Lymphatic Insufficiency as a Cause of Diastolic Dysfunction

A major advance in the field was the demonstration, in a direct experimental model, that cardiac lymphatic insufficiency itself can generate a heart-failure-like phenotype. Pu et al. showed that lymphatic insufficiency leads to diastolic dysfunction accompanied by edema, inflammation, fibrosis, and hypertrophy [31]. This finding is conceptually important because it suggests that lymphatic dysfunction is not merely a secondary modifier of established disease but can itself become a driver of myocardial dysfunction.

This framework is especially relevant to disease states dominated by stiff ventricles and interstitial remodeling. Diastolic performance depends strongly on matrix composition, interstitial pressure, and capillary-interstitial coupling. Persistent failure of myocardial drainage may therefore promote a state of chronic interstitial overload that progressively reduces compliance and impairs reserve [22,23,31].

## 8. Cardiac Lymphatics as the Link Between Congestion and Inflammation

Perhaps the most important contribution of this paradigm is that it links congestion and inflammation in a single biological framework. Congestion is usually discussed as a hydrostatic and renal problem, whereas inflammation is usually framed as an immunologic one [4,5,6,7,8,9,10,11,12]. Cardiac lymphatic dysfunction suggests that these processes are tightly interconnected.

Elevated venous pressure increases capillary filtration and promotes interstitial fluid accumulation. At the same time, venous hypertension impairs the gradient required for lymph to return to the venous circulation [4,19,20,21,33]. The consequence is persistent tissue congestion. Because this retained interstitial fluid contains proteins, cytokines, and immune mediators, tissue congestion is intrinsically inflammatory.

This generates a self-amplifying loop: edema promotes inflammatory persistence; inflammation and matrix remodeling impair lymphatic competence; reduced lymphatic drainage worsens edema and cytokine retention; and the cycle culminates in fibrosis and chamber stiffening [19,20,21,24,31,33,34]. This concept helps explain why some patients remain symptomatic despite apparently successful vascular decongestion. Standard diuretic treatment may improve intravascular volume but does not necessarily normalize interstitial protein clearance, immune-cell egress, or tissue-level fluid homeostasis [4,5,20,21,34].

**Figure 2:**
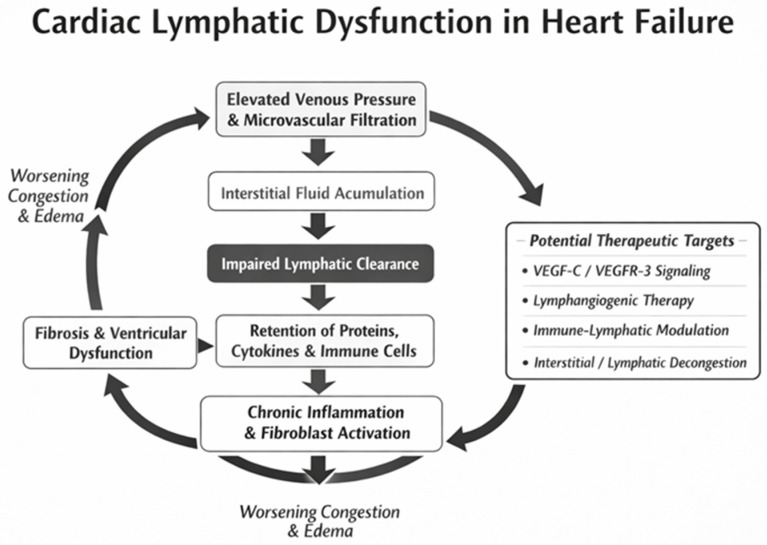
Cardiac lymphatic dysfunction as a mechanistic bridge between congestion and inflammation in heart failure. Elevated venous pressure and increased microvascular filtration promote myocardial interstitial fluid accumulation. When lymphatic clearance becomes insufficient, proteins, cytokines, and immune cells are retained within the myocardium, sustaining chronic inflammation and fibroblast activation. This leads to fibrosis, ventricular dysfunction, and worsening tissue congestion, thereby establishing a self-amplifying cycle. Potential therapeutic targets include VEGF-C/VEGFR-3 signaling, lymphangiogenic therapy, immune-lymphatic modulation, and interstitial or lymphatic decongestion strategies.

A conceptual framework linking venous congestion, impaired lymphatic clearance, inflammatory persistence, fibrosis, and ventricular dysfunction is shown in Figure 2.

The principal mechanisms through which cardiac lymphatic dysfunction may contribute to heart failure are summarized in Table 1.

## 9. Relevance to Heart Failure Phenotypes

The strongest mechanistic evidence for lymphatic involvement comes from preclinical models of ischemic injury and post-MI HFrEF-like remodeling [25,26,27,28,29,30,45,46,47,48,49,50]. In this setting, a mismatch between inflammatory burden and lymphatic clearance worsens edema resolution and adverse remodeling.

The concept may be particularly relevant in HFpEF. Paulus and Tschöpe proposed that HFpEF is driven in part by coronary microvascular endothelial inflammation [52]. Cuijpers et al. extended this framework by highlighting the role of microvascular and lymphatic dysfunction in HFpEF and its associated comorbidities [53]. Rossitto et al. further reported reduced lymphatic reserve in obese HFpEF, supporting the concept that tissue-fluid handling is abnormal in this phenotype [35].

This is biologically plausible because HFpEF is associated with obesity, diabetes, aging, endothelial dysfunction, chronic low-grade systemic inflammation, increased venous pressures, and diffuse interstitial remodeling. These abnormalities may increase capillary permeability and interstitial fluid load while simultaneously reducing lymphatic reserve. In obesity-associated HFpEF, impaired lymphatic reserve may amplify tissue congestion, delay clearance of inflammatory mediators, and contribute to myocardial stiffening. Cardiac lymphatic dysfunction may therefore represent one mechanism linking systemic inflammation, microvascular dysfunction, obesity-related congestion, myocardial stiffness, and congestion-related symptoms and signs in HFpEF.

## 10. Diagnostics: Toward a Lymphatic Phenotype of Heart Failure

Current HF diagnostics do not directly measure cardiac lymphatic function. Clinical examination, natriuretic peptides, echocardiography, and hemodynamic measurements remain indispensable, but they do not define myocardial interstitial drainage [1,2,4,20,21]. This may obscure a clinically relevant disease mechanism.

Cardiac magnetic resonance provides one indirect window into this problem. Myocardial edema, extracellular volume expansion, and fibrosis imaging may all reflect consequences of impaired lymphatic clearance [22,23]. Although these methods are not lymphatic-specific, they may help identify phenotypes characterized by disproportionate interstitial pathology.

Circulating markers of lymphangiogenic signaling may also prove useful. Wada et al. showed distinct prognostic characteristics of VEGF-D and VEGF-C in coronary disease [54]. While not specific to myocardial lymphatic function, such markers illustrate the potential for biomarker-assisted phenotyping. Over time, a clinically meaningful “lymphatic-interstitial congestion” phenotype may emerge alongside more traditional hemodynamic classifications.

However, important limitations remain. Cardiac magnetic resonance can detect myocardial edema, extracellular volume expansion, and fibrosis, but these findings are not specific to lymphatic dysfunction and may also reflect inflammation, ischemia, loading conditions, or extracellular matrix remodeling. Similarly, circulating VEGF-C, VEGF-D, and related lymphangiogenic markers are not cardiac-specific and may be influenced by systemic inflammation, vascular disease, renal function, obesity, and extracardiac lymphatic biology. Direct imaging of cardiac lymphatic transport remains technically challenging and is not currently validated for routine HF phenotyping. Consequently, a clinically applicable lymphatic phenotype of HF will require standardized imaging protocols, validated biomarkers, and correlation with functional measures of tissue-fluid handling.

## 11. Therapeutic Implications

Current HF therapy was not designed to restore myocardial lymphatic transport. Guideline-directed therapy reduces neurohormonal activation, afterload, sodium retention, and adverse remodeling [1,2], but does not directly normalize lymphatic drainage or immune-cell clearance.

The most mature preclinical experimental strategy is therapeutic lymphangiogenesis. VEGF-C-based stimulation of lymphatic growth improves edema resolution, reduces fibrosis, and enhances functional recovery after MI [25,26]. Similar benefits have been observed in preclinical pressure-overload models [29,30]. Song et al. further showed that lymphangiogenic therapy can prevent cardiac dysfunction by ameliorating inflammation and hypertension [55].

The translational rationale for this approach has been reinforced by recent reviews [56,57,58,59,60]. These works emphasize that future strategies may need to improve not only vessel number but also lymphatic maturation, valve competence, transport function, and immune-regulatory signaling.

Device-based or drainage-based approaches are also emerging. Martens et al. proposed targeting the lymphatic system for interstitial decongestion [34]. Biegus et al. described the rationale for thoracic duct-based decongestion in acute HF [61], and Salah et al. later reported an early clinical feasibility study of lymphatic drainage in patients with HF [32]. These approaches remain early and investigational but are conceptually important because they redefine decongestion as restoration of tissue-fluid clearance rather than simply removal of plasma volume.

More broadly, contemporary HF management is increasingly moving toward phenotype-tailored approaches, including advanced device-based therapies in selected chronic HF populations, reinforcing the need to define biologically meaningful subgroups that may benefit from emerging interventions [62,63].

Although these findings provide a strong mechanistic rationale, their clinical translation remains investigational. At present, lymphangiogenic therapy and lymphatic-drainage approaches should not be interpreted as established HF treatments. Key unresolved issues include patient selection, timing of intervention, safety of sustained lymphangiogenic stimulation, durability of lymphatic repair, and the identification of clinically meaningful endpoints. Therefore, these strategies should be considered hypothesis-generating until validated in adequately powered human studies.

The main experimental and translational evidence supporting the role of cardiac lymphatics in heart failure is summarized in Table 2.

## 12. Challenges and Future Directions

Several challenges remain before cardiac lymphatic dysfunction can be integrated into routine HF practice. First, lymphatic vessel abundance must be distinguished from lymphatic function. Histological lymphangiogenesis is not equivalent to effective transport competence [29,51]. Second, more human data are needed. Most mechanistic insights still come from animal studies, whereas human evidence is currently limited to observational, translational, and early feasibility studies. Third, the timing and reversibility of lymphatic dysfunction across HF stages remain uncertain.

Overall, the current evidence base remains uneven. Mechanistic support is strongest in preclinical models of myocardial infarction, pressure overload, and direct lymphatic insufficiency. Future work should focus on validating cardiac lymphatic dysfunction in well-phenotyped HF populations, defining clinically measurable lymphatic phenotypes, and testing whether lymphatic-restorative interventions improve clinically relevant outcomes.

Different HF phenotypes are also likely to have distinct lymphatic signatures. Post-infarction HFrEF, pressure-overload disease, obesity-related HFpEF, and right-sided congestive states are unlikely to share identical biology. Finally, future work must integrate cardiac lymphatics into broader systems-level models of congestion that include venous pressure, endothelial permeability, renal function, and tissue tolerance.

## 13. Conclusions

Cardiac lymphatic dysfunction is emerging as a coherent and clinically relevant paradigm in HF. The cardiac lymphatic circulation regulates interstitial fluid balance, immune-cell trafficking, inflammatory resolution, and matrix homeostasis. When this system becomes insufficient, the result may be persistent myocardial edema, inflammatory persistence, fibrosis, and ventricular dysfunction.

This framework is especially valuable because it links congestion and inflammation, two defining features of HF that are often treated separately. Preclinical experimental evidence strongly supports a role for lymphatic dysfunction in infarct healing, pressure-overload remodeling, and diastolic dysfunction. Human data remain more limited and consist mainly of observational, translational, and early feasibility evidence. Accordingly, lymphatic-restorative and interstitial decongestion strategies should currently be viewed as promising but investigational approaches that require prospective clinical validation.

The failing heart should therefore be understood not only as a mechanically stressed pump, but also as an organ with disturbed lymphatic homeostasis. Recognizing that disturbance may open a new frontier in HF phenotyping and therapy.

## Figures and Tables

**Table 1:** Major mechanisms by which cardiac lymphatic dysfunction may contribute to heart failure.

Pathophysiological Domain	Proposed Role of Cardiac Lymphatic Dysfunction	Consequence in Heart Failure
Interstitial fluid handling	Reduced clearance of myocardial ultrafiltrate	Persistent myocardial edema and tissue congestion
Protein and macromolecule removal	Retention of proteins, proteases, and matrix-active mediators within the interstitium	Increased interstitial oncotic load, matrix disruption, and sustained tissue swelling
Immune-cell trafficking	Impaired leukocyte egress and delayed clearance of inflammatory cells and debris	Persistent sterile inflammation and defective inflammatory resolution
Fibro-inflammatory signaling	Prolonged retention of cytokines and immune mediators with sustained fibroblast activation	Interstitial and perivascular fibrosis, ventricular stiffening, and maladaptive remodeling
Microvascular-interstitial coupling	Increased capillary compression and impaired diffusion associated with unresolved edema	Reduced oxygen delivery, impaired myocardial efficiency, and worsening dysfunction
Hemodynamic interaction	Venous hypertension reduces the pressure gradient for lymph return and further impairs drainage	Amplification of congestion despite conventional decongestive therapy
Ventricular remodeling	Chronic edema and inflammatory persistence promote extracellular matrix expansion and chamber dysfunction	Progressive systolic and/or diastolic impairment
Therapeutic implication	Lymphatic insufficiency identifies a potential target beyond intravascular volume reduction	Rationale for tissue-focused decongestion and lymphatic-restorative therapies

**Table 2:** Experimental and translational evidence supporting the role of cardiac lymphatics in heart failure.

Clinical/Experimental Setting	Main Lymphatic Observation	Functional Implication	Key References
Myocardial infarction	Endogenous lymphangiogenesis is activated after injury but is frequently insufficient relative to tissue demand	Persistent edema, delayed immune-cell clearance, increased fibrosis, and adverse remodeling when lymphatic adaptation is inadequate	[25,26,27,28,47,48,49,50]
Therapeutic lymphangiogenesis after myocardial infarction	VEGF-C-driven or related lymphatic stimulation enhances lymphatic remodeling	Improved edema resolution, reduced fibrosis, improved inflammatory resolution, and better ventricular recovery	[25,26,28,49,55,56,57,58,59,60]
Pressure-overload remodeling	Cardiac lymphatic remodeling occurs during chronic hemodynamic stress	May limit inflammation and perivascular fibrosis, but functional adequacy is not guaranteed	[29,30,51]
Direct lymphatic insufficiency	Loss of cardiac lymphatic competence can itself induce myocardial edema, fibrosis, and hypertrophy	Diastolic dysfunction and heart-failure-like remodeling may develop even without primary infarction	[31]
HFpEF and obesity-related congestion	Reduced lymphatic reserve and combined microvascular-lymphatic dysfunction have been proposed	Chronic tissue congestion, impaired reserve, and diffuse interstitial remodeling may be promoted	[35,52,53]
Human heart failure and translational studies	Lymphatic dysregulation has been recognized as a clinically relevant component of congestion biology	Supports development of new phenotyping strategies and interstitial decongestion approaches	[20,21,32,34,61,62]
Interventional lymphatic decongestion	Thoracic duct or related lymphatic drainage strategies are under early investigation	Potential adjunct to standard diuretic-based decongestion in selected patients	[32,34,61,62]

## Data Availability

The original contributions presented in this study are included in the article. Further inquiries can be directed to the corresponding author.
